# 

**Published:** 1857-03

**Authors:** 


### RECEIPTS ON SUBSCRIPTIONS,

Up to March 10th, 1857.

### ILLINOIS.

| | | |
|---|---|---|
| Dr. M. Parker, | Vol. 6, | $2.00 |
| " J. Bowman, | " 6, | 1.00 |
| " W. P. Short, | " 6, | 2.00 |
| " J. W. Wilkinson, | " 5, | 2.00 |
| " J. B. Paul, | " 6, | 2.00 |
| " E. F. Hubbard, | " 6, | 2.00 |
| " Isaac Rice, | " 6, | 2.00 |
| " G. W. Albin, | " 6, | 2.00 |
| " J. Leeds, | " 5, | 2.00 |
| " Sam'l McNair, | " 6, | 2.00 |
| " S. L. Urmston, | " 6, | 2.00 |
| " O. S. Johnson, | Vol. 5 & 6, | 4.00 |
| " Chas. Hill, | Vol. 6, | 2.00 |
| " W. M. Hall, | " 6, | 2.00 |
| " Geo. J. Sweet, | " 6, | 2.00 |
| " B. S. Corey, | " 6, | 2.00 |
| " Thos. Bevan, | " 6, | 2.00 |
| " L. Brookhart, | " 6, | 1.00 |
| " E. K. Farmer, | Vol. 5 & 6, | 4.00 |
| " J. Vandyke, | Vol. 6, | 2.00 |
| " John Wright, | " 5, | 2.00 |
| " T. J. Shreves, | " 5, | 2.00 |
| " J. C. Lowrie, | " 6, | 2.00 |
| " F. Erhardt, | " 5, | 2.00 |
| " Mergler, | " 5, | 1.00 |
| " Robinson, | " 6, | 2.00 |
| " J. C. Patterson, | Vol. 5 & 6, | 4.00 |
| " Alex. Taylor, | Vol. 6, | 2.00 |
| " John Crim, | " 6, | 2.00 |
| " S. M. Reynolds, | " 6, | 1.00 |
| " T. L. Catherwood, | " 5, | 2.00 |

### INDIANA.

| | | |
|---|---|---|
| Dr. J. H. Harriman, | Vol. 6, | $2.00 |
| " A. W. Dewey, | " 6, | 2.00 |
| " Jefferson Miller, | " 6, | 2.00 |
| " Samuel Doggy, | " 6, | 2.00 |
| " A. J. Mirick, | " 5, | 2.00 |
| " W. F. Green, | " 6, | 2.00 |
| " J. Gregory, | " 6, | 2.00 |
| " L. D. Personnett, | " 6, | 2.00 |
| " J. P. Terrell, | " 6, | 2.00 |
| " T. A. Graham, | " 6, | 2.00 |
| " J. Y. Campbell, | " 6, | 2.00 |
| " J. W. Hall, | " 6, | 2.00 |
| " N. Sherman, | Vol. 3, 4, 5, 6 | 7.00 |
| " R. M. Gilkeson, | Vol. 5, | 2.00 |

### WISCONSIN.

| | | |
|---|---|---|
| Drs. Thayer & Pashley, | Vol. 6, | $2.00 |
| " S. Blood, | " 5, | 2.00 |
| " Cooper & Darling, | " 6, | 1.00 |
| " J. B. Moffett, | " 5, | 2.00 |
| " H. Wearne, | " 6, | 2.00 |

### IOWA.

| | | |
|---|---|---|
| Dr. Joseph Casto, | Vol. 6, | $2.00 |
| " J. Williamson, | " 5, | 2.00 |
| " J. W. Cowden, | " 6, | 2.00 |

### MICHIGAN.

| | | |
|---|---|---|
| Dr. N. A. Hill, | Vol. 6, | $2.00 |

### OHIO.

| | | |
|---|---|---|
| Dr. Thos. P. Bond, | Vol. 5, | $2.00 |

### CALIFORNIA.

